# The effect of the 2009 revised U.S. guidelines for gestational weight gain on maternal and infant health: a quasi-experimental study

**DOI:** 10.1186/s12884-023-05425-8

**Published:** 2023-02-17

**Authors:** Daniel F. Collin, Richard Pulvera, Rita Hamad

**Affiliations:** 1 Philip R. Lee Institute for Health Policy Studies, University of California San Francisco, 995 Potrero Avenue, Building 80, Ward 83, San Francisco, CA 94110 USA; 2 School of Public Health, University of California Berkeley, Berkeley, CA USA; 3 Department of Family & Community Medicine, University of California San Francisco, 995 Potrero Avenue, Building 80, Ward 83, San Francisco, CA 94110 USA

**Keywords:** Gestational weight gain, Quasi-experimental studies, Maternal health, Infant health, Pregnancy Risk Assessment Monitoring System

## Abstract

**Background:**

Excess gestational weight gain (GWG) has adverse short- and long-term effects on the health of mothers and infants. In 2009, the US Institute of Medicine revised its guidelines for GWG and reduced the recommended GWG for women who are obese. There is limited evidence on whether these revised guidelines affected GWG and downstream maternal and infant outcomes.

**Methods:**

We used data from the 2004–2019 waves of the Pregnancy Risk Assessment Monitoring System, a serial cross-sectional national dataset including over 20 states. We conducted a quasi-experimental difference-in-differences analysis to assess pre/post changes in maternal and infant outcomes among women who were obese, while “differencing out” the pre/post changes among a control group of women who were overweight. Maternal outcomes included GWG and gestational diabetes; infant outcomes included preterm birth (PTB), low birthweight (LBW), and very low birthweight (VLBW). Analysis began in March 2021.

**Results:**

There was no association between the revised guidelines and GWG or gestational diabetes. The revised guidelines were associated with reduced PTB (− 1.19% points, 95%CI: − 1.86, − 0.52), LBW (− 1.38% points 95%CI: − 2.07, − 0.70), and VLBW (− 1.30% points, 95%CI: − 1.68, − 0.92). Results were robust to several sensitivity analyses.

**Conclusion:**

The revised 2009 GWG guidelines were not associated with changes in GWG or gestational diabetes but were associated with improvements in infant birth outcomes. These findings will help inform further programs and policies aimed at improving maternal and infant health by addressing weight gain in pregnancy.

**Supplementary Information:**

The online version contains supplementary material available at 10.1186/s12884-023-05425-8.

## Background

Excess or inadequate gestational weight gain (GWG) has adverse short- and long-term effects on the health of mothers and infants. In the short term, excess GWG has been associated with increased risk for hypertensive disorders in pregnancy (HDPs), Caesarean delivery, and infants born preterm or large-for-gestational-age (LGA), while inadequate GWG can lead to increased risk of small-for-gestational-age (SGA) infants [1, 2]. In the long term, excess GWG is associated with increased risk of postpartum weight retention for the mother, and overweight and obesity during childhood for the infant, which can lead to higher morbidity and mortality for both [3–5]. The effects of GWG on maternal and infant health are more salient among women who were overweight (i.e., body mass index [BMI] 25–29.9) or obese (i.e., BMI ≥ 30) before pregnancy [6–9]. Given that the prevalence of obesity among adults in the US has increased in the past decade, and pre-pregnancy obesity prevalence rose from 26.1% in 2016 to 29.0% in 2019, there is a need for policies and programs that target maternal nutrition and GWG to improve maternal and infant health [10, 11].

In the US, the Institute of Medicine (IOM, now the National Academy of Medicine) has historically issued and updated clinical guidelines on GWG intended to improve perinatal outcomes. First released in 1970, the IOM guidelines provided a range of recommended GWG for pregnant women that was the same regardless of a woman’s pre-pregnancy BMI (Table 1). The guidelines were revised in 1990, with the primary intention of increasing GWG and improving infant birthweight among underweight women, recommending different GWG ranges depending on four categories of maternal pre-pregnancy BMI [14]. The guidelines were again revised in 2009 to more specifically address the greater percentage of women entering pregnancy overweight or obese and having excess GWG [15]. The main change for the 2009 revised guidelines was to recommend that women who are obese gain even less weight during pregnancy than those who are overweight.

**Table 1:** Guidelines for recommended gestational weight gain, by pre-pregnancy body mass index

		*Pre-pregnancy Body Mass Index*	
		Overweight	Obese
*Version of* *IOM Guidelines*	1970 guideline	20–25 lbs	20–25 lbs
	1990 revision	15–25 lbs	≥15 lbs^a^
	2009 revision	15–25 lbs	11–20 lbs

Overweight (body mass index 25.0–29.9); Obese (body mass index ≥30.0)

*Abbreviations*: *IOM* Institute of Medicine

^a^While there was no maximum gestational weight gain recommended for women who were obese in the 1990 IOM guidelines, in practice it was considered to be similar to that for women who were overweight (i.e., 25 lbs) [12, 13].

While previous studies have examined the benefits of adhering to the 2009 revised guidelines, there is little research on the effects of the 2009 IOM revised guidelines on GWG or perinatal health outcomes among women who are obese. Of note, one study examining the effect of the 1990 revised guidelines found no effect on GWG among US women while another found a decrease in percentage of women within the recommended GWG [16, 17]. Our current study addresses this gap by examining the effects of the 2009 revision on several maternal and infant health outcomes in a large diverse multi-state sample. We hypothesized that the implementation of a lower range of recommended GWG for women who are obese would decrease GWG and improve related maternal and infant health outcomes.

## Methods

### Data

Data were drawn from the 2004–2019 waves of the Pregnancy Risk Assessment Monitoring System (PRAMS), a surveillance project of the US Centers for Disease Control and Prevention in conjunction with state and local public health entities. Detailed PRAMS methodology has been previously described [18]. Briefly, PRAMS includes a representative sample of women drawn from birth certificates from each participating site (state or territory) and collects survey responses on demographics and health outcomes before, during, and shortly after pregnancy, which are then linked with birth certificates. Each participating site samples between 1300 and 3400 women per year, and participating sites represent approximately 81% of all US live births.

### Sample selection

**Fig. 1:**
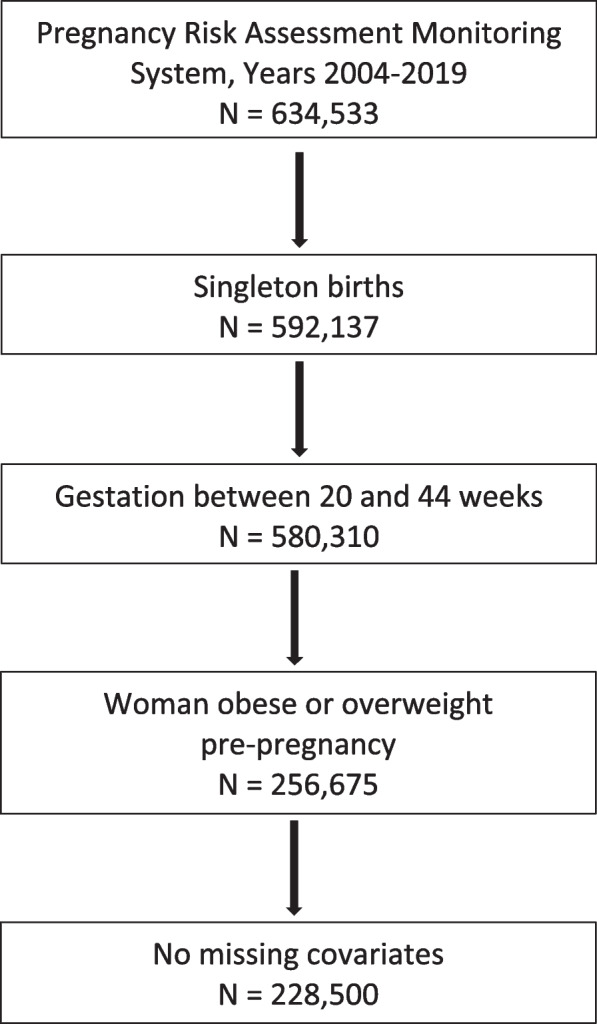
Sample Selection. Pregnancy Risk Assessment Monitoring System (PRAMS) dataset includes linked birth certificate and women’s survey responses. Main analysis was restricted to women with live-born singleton births with a gestational age of 20–44 weeks at delivery in states for which PRAMS makes data available and whose pre-pregnancy weight was categorized as obese (body mass index ≥30) or overweight (body mass index 25.0–29.9)

We used PRAMS survey waves 2004–2019 (*N* = 634,533). Data prior to 2004 were excluded due to differences in how birth certificate data were collected, and 2019 was the most recent year of data available at the start of our analyses in March 2021. We included women with live-born singleton births with a gestational age of 20–44 weeks at delivery in states for which PRAMS makes data available. The sample included women whose pre-pregnancy weight was categorized as obese, representing the “treatment” group in the analysis described below, and those categorized as overweight, representing the “control” group. We excluded women with underweight and normal pre-pregnancy BMI, since they may differ from women who are obese in important ways. The final sample size was 228,500 (Fig. 1, sample flowchart).

Notably, the 2009 revised guidelines defined BMI using categories based on the World Health Organization (WHO) categories, while the older IOM guidelines used categories based on the Metropolitan Life Insurance tables. So that any results are not driven by changes in the composition of the treatment and control groups due to the changes in the definition of these categories, we used the WHO BMI categories for observations that occurred before and after the revision to maintain consistency in the treatment and control groups [15].

### Exposure

The primary exposure was a dichotomous variable indicating whether the pregnancy took place after the revised IOM GWG guidelines, which were released in May 2009. We considered pregnancies to occur during the post-revision period if the birth occurred on or after 1 July 2010, allowing 6 months for guidelines to be disseminated and implemented in prenatal care and a 9-month pregnancy. This parallels the approach in a previous study that examined the 1990 IOM guidelines [16]. This definition was varied in secondary analyses, described below.

### Outcomes

We selected maternal and infant outcomes that could be affected by changes to maternal nutrition and GWG. Maternal outcomes included GWG (continuous, from linked birth certificates), and gestational diabetes (binary, from survey self-report and birth certificates).

Infant outcomes included preterm birth (PTB, < 37 weeks’ gestation), low birthweight (LBW, < 2500 g), very low birthweight (VLBW, < 1500 g), SGA, LGA, and macrosomia (birthweight ≥4500 g). While analyses using continuous outcomes would improve statistical power, PRAMS provides data on infant outcomes as categorical variables, and we analyzed these as binary variables for ease of interpretation.

### Covariates

Potential confounders included as model covariates were women’s age, race/ethnicity, education, marital status, parity, Medicaid used for prenatal care, household income in the year prior to delivery, and indicator variables for delivery year.

### Analysis

First, we calculated sample characteristics for women who were overweight or obese before and after the 2009 guidelines. Next, our analysis leveraged the fact that the 2009 guidelines resulted in a reduction in the recommended GWG for women who are obese, but no changes for women who are overweight (Table 1). We therefore estimated the effects of the revised guidelines using a difference-in-differences (DID) approach. DID is a quasi-experimental technique well suited to examining the effects of policies and programs while accounting for secular (i.e., underlying longitudinal) trends in outcomes [19, 20]. It estimates the change in the outcome in the treatment group (in this case, women who were obese and subject to a change in recommended GWG) before and after the intervention, while subtracting or “differencing” out the change in the outcome in the control group (in this case, who were overweight and not subject to a change in recommended GWG). As noted above, we defined births to occur during the post-revision period if they took place on or after 1 July 2010.

In practice, DID involves regressing each outcome on an interaction term between a binary variable for whether a pregnancy occurred before versus after the revised guidelines, and a binary variable for whether a woman’s pre-pregnancy BMI was categorized as overweight versus obese. As is standard in DID analysis, we used multivariable linear regressions to analyze both binary and continuous outcomes due to the differences in the interpretation of interaction terms in non-linear models [21, 22]. The coefficients for binary outcomes can therefore be interpreted as percent change in risk (i.e., a linear probability model). See Supplemental Methods for details.

### DID assumptions

DID analysis rests on several assumptions, including the “parallel trends” assumption that trends in the outcomes were similar in the treatment and control groups during the pre-revision period, and that observed effects are not driven by differential compositional changes in the two groups. We evaluated the validity of these assumptions using standard techniques, described in the Supplement.

### Secondary analyses

We conducted subgroup analyses to examine whether there were heterogeneous effects by key covariates, since it may be that different subgroups of women (or their providers) faced structural, clinical, or personal barriers to implementing the guidelines. To do so, we stratified DID analyses by education, race/ethnicity, age, and parity. For education, we estimated the effects separately for high school or less and more than high school. For race/ethnicity we combined Asian/Pacific Islander, American Indian/Alaskan Native, and other non-White/mixed race individuals into a single category due to small cell sizes that could lead to unstable estimates. To investigate whether estimates from these stratified models were statistically significantly different from one another, we also conducted analyses including an interaction term between the main exposure variable and each covariate of interest.

We also conducted a secondary analysis in which we defined a pregnancy as occurring during the post-revision period if the birth occurred on or after 1 October 2010, allowing an additional 3 months of implementation of the guidelines into prenatal care. Additional secondary analyses are described in the Supplement.

## Results

### Sample characteristics

Women who were obese were more likely to have less education, be unmarried, be recipients of Medicaid or Special Supplemental Nutrition Program for Women, Infants, and Children (WIC) during pregnancy, and have lower income compared to women who were overweight during the same period (Table 2). They also had lower GWG on average, and were more likely to have gestational diabetes and infants that were preterm, LBW, VLBW, LGA, and macrosomic. Of note, DID assumes that the trends (i.e., slopes) in the outcomes, not the levels, are similar.

**Table 2:** Sample characteristics by revision period and pre-pregnancy weight category

	Pre July 2010		Post July 2010	
	Overweight ***N*** = 47,135	Obese ***N*** = 41,913	Overweight ***N*** = 70,346	Obese ***N*** = 69,826
	% or Mean (SD)	% or Mean (SD)	% or Mean (SD)	% or Mean (SD)
**Maternal Characteristics**				
Age, years				
< 25	31.7	29.6	23.5	22.9
25–34	51.7	54.0	58.2	59.1
35+	16.6	16.4	18.3	18.0
Race/Ethnicity				
White	54.4	53.2	49.2	46.4
Black	18.7	23.2	18.2	23.6
Hispanic/Latina	14.4	12.4	17.7	16.8
Asian/Pacific Islander	6.3	4.0	6.2	3.1
American Indian/Alaskan Native	4.4	5.4	3.9	4.9
Other	1.8	1.8	4.8	5.3
Education				
Less than high school	14.7	14.2	11.7	11.7
High school	30.3	34.8	24.0	28.8
Some college	27.2	30.3	31.1	36.1
College or more	27.8	20.6	33.2	23.5
Married	62.8	60.3	61.2	55.5
Parity				
Nulliparous	39.3	36.9	37.6	35.1
Parity 1	31.7	31.4	32.0	31.2
Parity 2+	29.0	31.7	30.5	33.8
Medicaid during pregnancy	43.7	50.7	44.7	53.6
Annual household income ≥ $50,000^a^	44.4	37.4	43.9	35.2
WIC during pregnancy	47.4	55.8	44.7	53.5
**Maternal Outcomes**				
Gestational weight gain, lbs	29.6 (14.7)	24.0 (15.8)	29.9 (15.4)	24.1 (17.0)
Gestational diabetes	11.6	18.3	11.4	18.1
GWG within IOM recommendation	28.8	27.3	26.2	24.8
**Infant Outcomes**				
Preterm birth	21.6	25.4	17.1	19.8
Low birthweight	24.2	27.8	18.4	20.7
Very low birthweight	6.0	8.6	3.6	4.9
Small for gestational age	14.5	14.8	13.3	12.7
Large for gestational age	11.0	13.5	10.8	14.0
Macrosomia	1.3	2.1	1.3	1.9

Sample was drawn from PRAMS participating states from 2004 to 2019 and included women with live-born singleton births with a gestational age of 20–44 weeks at delivery and whose pre-pregnancy weight was categorized as obese (BMI ≥ 30.0) or overweight (BMI 25.0–29.9)

*Abbreviations*: *BMI* Body mass index, *GWG* Gestational weight gain, *IOM* Institute of Medicine, *PRAMS* Pregnancy Risk Assessment Monitoring System, *WIC* Special Supplemental Nutrition Program for Women, Infants, and Children

^a^Inflation adjusted to 2018 US dollars

### Tests of DID assumptions

Analyses revealed that SGA, LGA, and macrosomia violated DID assumptions (eTable 1 and eFigure 2), implying that women who were overweight were not an appropriate control group for women who were obese for these outcomes. These were therefore excluded from the results below. Additional details on tests of DID assumptions are described in the Supplement.

### Effects of revised IOM guidelines

In the overall sample, we were unable to rule out the null hypothesis that the 2009 revised IOM guidelines had no effect on GWG or gestational diabetes among women who were obese, although the coefficient was negative in the main analysis and all other models (Table 3). There were, however, reductions in PTB (− 1.19% points, 95%CI: − 1.86, − 0.52), LBW (− 1.38% points, 95%CI: − 2.07, − 0.70), and VLBW (− 1.30% points, 95%CI: − 1.68, − 0.92).

**Table 3:** Effect of 2009 IOM revised GWG guidelines on maternal and infant outcomes

	Effect of IOM Revised Guidelines (95% CI)	
	Main Analysis: Post Period July 2010	Secondary analysis: Post Period October 2010
Gestational weight gain, lbs	−0.20	−0.19
	(−0.47, 0.07)	(−0.46, 0.08)
Gestational diabetes	0.09	0.32
	(−0.50, 0.68)	(−0.27, 0.90)
Preterm birth	−1.19*	−1.27*
	(−1.86, − 0.52)	(−1.94, − 0.61)
Low birthweight	−1.38*	−1.46*
	(−2.07, − 0.70)	(−2.14, −.78)
Very low birth weight	−1.30*	− 1.32*
	(− 1.68, − 0.92)	(− 1.69, − 0.95)

Values in table represent the coefficients on the interaction term between a binary variable for whether a pregnancy occurred during the post-period (i.e., on or after July 2010) and a binary variable for whether a woman’s pre-pregnancy body mass index was categorized as overweight versus obese. Coefficients for binary outcomes were multiplied by 100 and therefore represent a change in percentage points. Analysis involved multivariable linear models (i.e., linear probability models for binary outcomes). Covariates included women’s age, race/ethnicity, education, marital status, insurance for prenatal care, parity, household income in the year prior to delivery, and delivery year

*Abbreviations*: *GWG* Gestational weight gain, *IOM* Institute of Medicine

* *p* < 0.05

### Secondary analyses

Subgroup analyses demonstrated heterogeneous effects by race/ethnicity and age (eFigures 4, 5, 6, 7 and 8). Black women who were obese experienced reduced GWG (− 0.77% points, 95%CI: − 1.41, − 0.14) and VLBW (− 2.22% points, 95%CI: − 3.25, − 1.18), and the latter was statistically significantly different from the estimate for White women (− 0.88 percentage points, 95%CI: − 1.38, − 0.37). Older women who were obese experienced reduced gestational diabetes risk (− 1.70% points, 95%CI: − 3.40, − 0.01), which was statistically significantly different from that among younger women (0.36% points, 95%CI: − 0.26, 0.98).

When we set the post period to be on or after 1 October 2010, results were similar to the main analysis (Table 3).

## Discussion

This study is among the first to estimate the health effects of the 2009 IOM revised guidelines for GWG among women who are obese, using a large multi-state data set and a quasi-experimental design. The main analysis showed that the 2009 guidelines had no effect on GWG although we did observe reductions in PTB, LBW, and VLBW.

There are several explanations for the null findings for GWG, which are similar to previous null findings of the effects of the 1990 IOM revised guidelines on GWG [16]. First, the results may be due to inadequate dissemination of the 2009 guidelines. For example, a study examining clinicians’ knowledge of the 2009 guidelines nearly a year after their release found that more than 50% were unaware of the new guidelines and only 2.3% correctly identified the BMI cutoffs and recommended GWG [23]. This could prevent clinicians from providing adequate BMI-specific counseling on recommended GWG. Alternately, studies in the US and abroad consistently showed an increase in the prevalence of women who were overweight and obese during the pre-pregnancy period and an increase in excess GWG from 2009 to 2018, suggesting that underlying secular trends driven by other factors may have worked against the IOM recommendations [24–26]. Relatedly, recent work has also found that adherence to the 2009 guidelines is generally low, with only about one-third of women having GWG within the recommended guidelines [27]. Knowledge of GWG recommendations, pre-pregnancy weight status, and adiposity-related risks during pregnancy is especially low among women of low socioeconomic status, which could also lead to (or be caused by) obstacles in communication with providers [28].

Even if awareness of the guidelines were greater, interventions to prevent excess GWG particularly among women who are overweight or obese have mostly been unsuccessful [12, 29, 30]. One reason may be that pregnant patients with a higher BMI report more negative healthcare experiences that lead some patients to avoid or delay care, which could prevent them from receiving adequate advice on nutrition and GWG [31]. Furthermore, obese and overweight women are more likely to receive advice on GWG, although they are also more likely to be told incorrect GWG targets [32, 33]. Alternately, it may be that it is particularly difficult for women who are obese to alter their GWG given the narrow window of pregnancy and other structural and pre-existing behavioral challenges. Unfortunately, PRAMS does not ask about GWG knowledge, health behaviors, or these types of healthcare interactions, so we are unable to assess these possible mediating pathways.

Other types of upstream policies and programs may be more effective at addressing GWG and downstream perinatal outcomes. For example, revisions to improve the healthfulness of WIC food packages have improved dietary quality during pregnancy and improved perinatal health outcomes including GWG [34–36]. The Earned Income Tax Credit (EITC), the largest U.S. poverty alleviation program, has also been linked to improvements in maternal and infant health [37–41]. These programs are comprehensive and concrete, providing food vouchers and nutritional education in the case of WIC and financial support in the case of the EITC.

On the other hand, this study demonstrated improvements in infant outcomes. While we had hypothesized that the main pathway through which the revised guidelines would impact infant outcomes would have been improved GWG, there may be other pathways. For example, the guidelines could have led to an improvement in diet and exercise or more frequent healthcare visits that could have led to improvements in infant outcomes. The IOM committee also recommended more studies on dietary intake, physical activity, and other factors that might affect maternal and infant health. Given that women who are obese may also be at risk for other health conditions and may receive more counseling on nutrition and health behaviors, this may have contributed to improvements in infant health. Unfortunately, PRAMS does not consistently include variables during our study period that capture these possible mediating pathways; this can be a topic for future research in other data sets.

Meanwhile, subgroup analyses in this study demonstrated inconsistent results. While most subgroup analyses were null, Black women who were obese had statistically significantly lower VLBW after the 2009 guidelines compared with White women, and older women had lower risk of gestational diabetes compared to younger women. Minority women, and in particular Black women, are more likely to have lower GWG compared to White women [42, 43], although the risk is not significantly different for Black obese and overweight women compared with White women [42]. With respect to age, excess GWG has been associated with obesity later in life [44] while the observed reduced risk for diabetes among women who are older and obese may be the result of closer prenatal care.

Our study has several strengths and limitations. In terms of strengths, we employed a quasi-experimental method and a large serial cross-sectional national data set to examine an important national set of guidelines that have the potential to impact at-risk pregnant women. The large geographically diverse sample covering several states reduces the possibility that the analysis is underpowered and makes results more generalizable. In terms of limitations, there may be measurement error or reporting biases for the exposure or for covariates that were self-reported. For example, while GWG was captured on the birth certificate, pre-pregnancy BMI was calculated from the PRAMS questionnaire which occurred on average two months after birth. Similarly, some studies suggest that there is differential misclassification of pre-pregnancy BMI, although others show that self-report pre-pregnancy weight are in general accurate measures [45, 46]. There is also a possibility that confounding by other polices/practices co-occurring with the IOM guidelines may explain the improvements in infant outcomes. In other words, there may be factors that influence the outcomes among the treatment group of obese women differentially than the control group of overweight women. This is a limitation of all DID analyses. One analysis that we did to address this involved conducting the analysis separately for WIC recipients and non-recipients, given that revisions to WIC also occurred in 2009 and WIC recipients are more likely to be obese, and found that this policy did not contribute to confounding (see eFigure 8 and Supplement); however, confounding by other unmeasured contemporaneous events may still be possible, as with any DID analysis.

## Conclusions

This evaluation of a 2009 revision to the IOM guidelines for GWG suggests that the new guidelines did not have an effect on GWG or gestational diabetes. This may be due to lack of awareness among providers and expectant mothers or because of difficulties in changing GWG in such a short timeframe among obese women. The new guidelines were also associated with improvements in downstream infant outcomes, and future work could examine the potential mechanisms that could have contributed to those improvements in the absence of a change in GWG. This study highlights the need to formulate other interventions to improve GWG and downstream perinatal health beyond the current recommendations.

## Supplementary Information

**Additional file 1.**

## Data Availability

The data that support the findings of this study are available from PRAMS and researchers may submit a proposal to PRAMS for access to the data (https://www.cdc.gov/prams/prams-data/researchers.htm).

## Abbreviations

BMI: Body mass index
DID: Difference-in-differences
EITC: Earned Income Tax Credit
GWG: Gestational weight gain
HDP: Hypertensive disorders in pregnancy
IOM: Institute of Medicine
LBW: Low birthweight
LGA: Large-for-gestational-age
PRAMS: Pregnancy Risk Assessment Monitoring System
PTB: Preterm birth
SGA: Small-for-gestational-age
VLBW: Very low birthweight
WIC: Special Supplemental Nutrition Program for Women, Infants, and Children
