# Supplementary material for: The effect of the 2009 revised U.S. guidelines for gestational weight gain on maternal and infant health: a quasi-experimental study
Source: BMC Pregnancy Childbirth. 2023 Feb 17;23:118. doi: 10.1186/s12884-023-05425-8 (PMC9936770; doi:10.1186/s12884-023-05425-8)
Supplement: Supplementary file 1 — Additional file 1. [file 12884_2023_5425_MOESM1_ESM.pdf]

SUPPLEMENTAL MATERIALS

SUPPLEMENTAL METHODS

Difference-in-differences Analysis

The equation for the difference-in-differences (DID) model is as follows:

$$Y = \beta_0 + \beta_1 Post + \beta_2 Obese + \beta_3 (Post\ x\ Obese) + \beta_4\ Cov + \theta_t + \varepsilon$$

Here, *Y* is one of the maternal or infant outcomes. *Post* is a dummy variable indicating whether a pregnancy occurred during the period after the 2009 Institute of Medicine (IOM) guidelines for gestational weight gain had been released (i.e., on or after July 2010 in the main analysis). *Obese* is a dummy variable indicating whether a woman's pre-pregnancy body mass index was obese as opposed to overweight. β_3 is the coefficient of interest on the interaction between *Post* and *Obese*, and represents the estimated effect of the revised IOM guidelines. *Cov* is a vector for individual-level covariates. θ_t represents indicator variables for year of delivery, to control for secular changes, and ε is the error term.

To assess whether women who are overweight represented an appropriate control group for women who are obese, we graphically assessed whether the trends (i.e., slopes) of the outcomes between women who are obese versus overweight were parallel during the pre-revision period. This is a standard evaluation of the "parallel trends assumption" of DID analysis.[1, 2] We also assessed the parallel trends assumption quantitively by restricting the sample to the pre-revision period, and regressing each outcome on the interaction between a binary variable for obese versus overweight and a continuous variable representing the time difference between birth date and IOM guideline implementation date (in months). A null result for this analysis would

indicate no statistically significant difference in the pre-revision trends for women who are obese versus overweight, satisfying the parallel trends assumption.

DID also assumes that any observed effects are not due to differential compositional changes between treatment and control groups over time. We evaluated this assumption by regressing each covariate separately on the interaction between a binary variable for obese versus overweight and a binary variable for pre/post revision. Any statistically significant coefficient on the interaction term in these regressions would indicate differential compositional changes that may bias the results.

Additional Details on Covariates

For income, PRAMS reports different categories in different years; we harmonized these to create a binary variable indicating whether annual inflation-adjusted household income was $50,000 or more. Percent missingness among covariates varied from 0.01% (age) to 7% (income). We did not impute missing values since complete case analysis is not thought to result in bias at such low levels of missingness.[3]

Tests of DID Assumptions

During the pre-revision period, most outcomes demonstrated parallel trends in both qualitative and quantitative evaluations (eTable 1 and eFigures 1-2). The exceptions were for SGA, LGA, and macrosomia, which we excluded from further analyses. The test of differential compositional changes showed significant differences in composition over time between women who were obese versus overweight for several variables (eTable 2), although the magnitude of these

differences was small. We adjusted for these covariates to account for possible confounding, although of course, we cannot test or adjust for differential composition in unobserved characteristics. This is a limitation of all DID analyses.

Secondary Analyses

We conducted several additional analyses to test the robustness of results to alternative specifications. The U.S. Special Supplemental Nutrition Program for Women Infant and Children (WIC) was revised in 2009, and obese women in our sample were more likely to be WIC recipients than overweight women (Table 2). To rule out the possibility that our main analysis is confounded by this contemporaneous policy change, we therefore conducted the analysis separately for WIC recipients and non-recipients. Results of this secondary analyses were similar to the main analysis (eFigure 8).

Additionally, given that the American College of Obstetrics and Gynecology (ACOG) did not adopt the guidelines as standard of care until January 2013,[4] we conducted an analysis in which we set the post-revision period to begin in January 2013, and omitted the period between May 2009 and January 2013. Results of this analysis were similar to the main analysis (eTable 3).

Finally, we analyzed the outcome of GWG as a binary variable for whether women's GWG was either within, above, or below the recommended range for their pre-pregnancy BMI, rather than as a continuous variable. However, during the pre-revision period, this set of binary variables did

not demonstrate parallel trends upon qualitative and quantitative evaluations (eTable 1 and eFigure 3), so results for this analysis are not presented.

SUPPLEMENTAL REFERENCES

1. Basu S, Meghani A, Siddiqi A: **Evaluating the health impact of large-scale public policy changes: classical and novel approaches**. *Annual Review of Public Health* 2017, **38**:351-370.
2. Dimick JB, Ryan AM: **Methods for evaluating changes in health care policy: the difference-in-differences approach**. *JAMA* 2014, **312**(22):2401-2402.
3. Allison PD: **Missing Data**. In: *The SAGE Handbook of Quantitative Methods in Psychology.* edn. London: SAGE Publications Ltd; 2009: 72-90.
4. American College of Obstetricians and Gynecologists: **ACOG Committee opinion no. 548: weight gain during pregnancy**. *Obstetrics and gynecology* 2013, **121**(1):210-212.

eTable 1. Quantitative Evaluation of Parallel Trends Assumption, by Outcome

Outcomes	β (95% CI)
Gestational weight gain, lbs	-0.0038<br>(-0.013, 0.0054)
Gestational diabetes	0.000040<br>(-0.00017, 0.00025)
GWG within IOM recommendation	-0.000037<br>(-0.00031, 0.00024)
GWG above IOM recommendation	-0.000078<br>(-0.00038, 0.00022)
GWG below IOM recommendation	0.00012<br>(-0.00012, 0.00035)
Preterm birth	0.000018<br>(-0.00023, 0.00027)
Low birthweight	0.000011<br>(-0.00025, 0.00027)
Very low birthweight	-0.00012<br>(-0.00027, 0.000034)
Small for gestational age	-0.00011<br>(-0.00032, 0.000097)
Large for gestational age	0.000064<br>(-0.00013, 0.00026)
Macrosomia	0.000034<br>(-0.000041, 0.00011)

Note: Data drawn from the Pregnancy Risk Assessment Monitoring System (PRAMS). Coefficient above represents the interaction between a binary variable for obese versus overweight and a continuous variable representing the time difference between birth date and post-period start date of July 2010 (in months). Analysis involved multivariable linear models (i.e., linear probability models for binary outcomes), covariates in the model included a binary variable for obese versus overweight, a continuous variable for time difference between birth date and post-period, and a variable representing the interaction between these two variables. Abbreviations: GWG, gestational weight gain; IOM Institute of Medicine

eTable 2. Test of Differential Compositional Changes

	**β**	**(95% CI)**
Age, years		
<25	0.015*	(0.0077, 0.022)
25-34	-0.014*	(-0.022, -0.0058)
35+	-0.00094	(-0.0073, 0.0055)
Race		
White	-0.016*	(-0.025, -0.0078)
Black	0.0094*	(0.0026, 0.016)
Hispanic/Latina	0.011*	(0.0045, 0.017)
Asian/Pacific Islander	-0.0087*	(-0.012, -0.0051)
American Indian/Alaskan Native	-0.00022	(-0.0037, 0.0033)
Other race/ethnicity	0.0051*	(0.0019, 0.0084)
Education		
Less than high school	0.0049	(-0.00071, 0.011)
High school	0.0020	(-0.0056, 0.0096)
Some college	0.018*	(0.010, 0.026)
College +	-0.025*	(-0.032, -0.017)
Married	-0.031*	(-0.039, -0.023)
Parity		
Nulliparous	-0.00099	(-0.0091, 0.0071)
Parity 1	-0.0053	(-0.013, 0.0026)
Parity 2+	0.0063	(-0.0016, 0.014)
Medicaid during pregnancy	0.019*	(0.010, 0.027)
Annual household income ≥ $50,000	-0.017*	(-0.025, -0.0088)

* p<0.05

Note: Data drawn from the Pregnancy Risk Assessment Monitoring System (PRAMS). Coefficients represent the interaction between the interaction between a binary variable for obese versus overweight women and a binary variable for pre/post Institute of Medicine revision period. Analysis involved multivariable linear models (i.e., linear probability models for binary outcomes). These models examine whether there are differential pre/post differences in the demographic characteristics between obese and overweight women. A null result indicates no differential pre/post trends in sample composition for a given covariate.

eTable 3. Effect of 2009 IOM Revised GWG Guidelines on Maternal and Infant Outcomes, Omitting May 2009-January 2013

	Effect of IOM Revised Guidelines (95% CI)
Gestational weight gain, lbs	-0.26
	(-0.57, 0.041)
Gestational diabetes	0.19
	(-0.47, 0.85)
Preterm birth	-0.97*
	(-1.73, -0.22)
Low birthweight	-1.50*
	(-2.26, -0.73)
Very low birth weight	-1.49*
	(-1.91, -1.07)

* $p<0.05$
Note: Values in table represent the coefficients on the interaction term between a binary variable for whether a pregnancy occurred during the post-period (i.e., on or after February 2013 versus prior to May 2009) and a binary variable for whether a woman's pre-pregnancy body mass index was categorized as overweight versus obese. Coefficients for binary outcomes were multiplied by 100 and therefore represent a change in percentage points. Analysis involved multivariable linear models (i.e., linear probability models for binary outcomes). Covariates included women's age, race/ethnicity, education, marital status, insurance for prenatal care, parity, household income in the year prior to delivery, and delivery year.
Abbreviations: GWG, gestational weight gain; IOM, Institute of Medicine

eFigure 1. Graphs of Trends for Maternal Outcomes

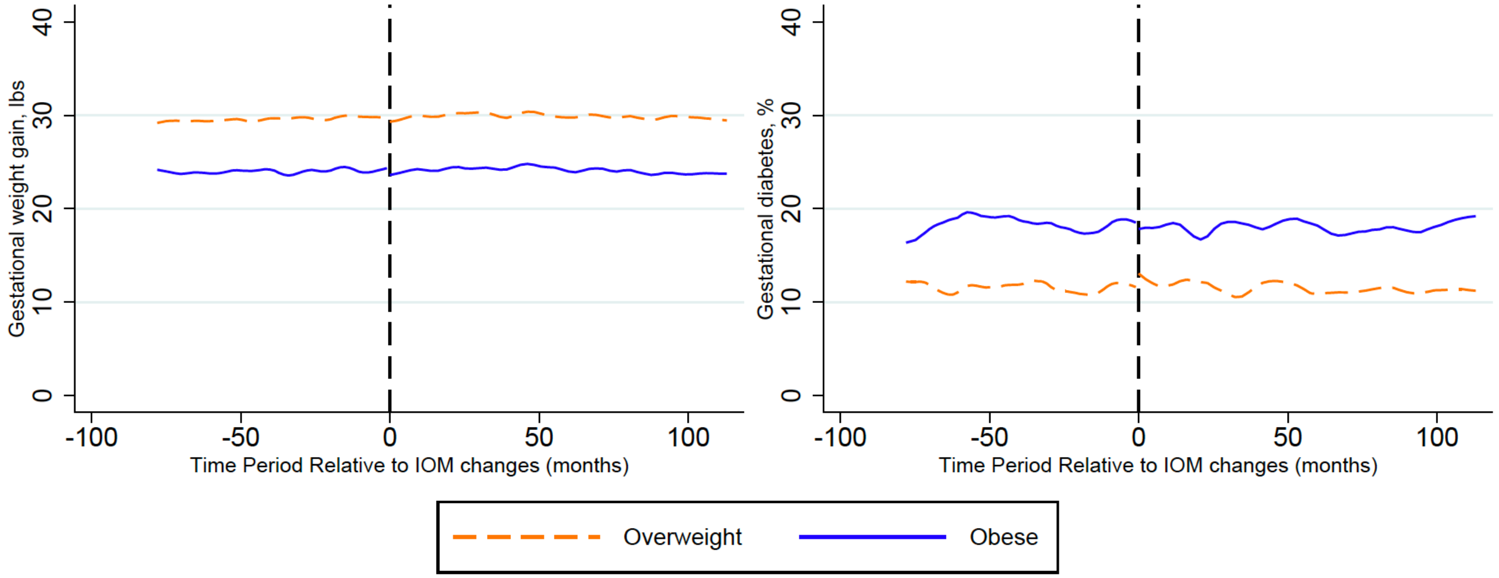

Note: Data drawn from the Pregnancy Risk Assessment Monitoring System (PRAMS). Time zero corresponds to July 1 2010.
Overweight (body mass index 25-29.9), obese (body mass index ≥30.0)
Abbreviations: IOM, Institute of Medicine

eFigure 2. Graphs of Trends for Infant Outcomes

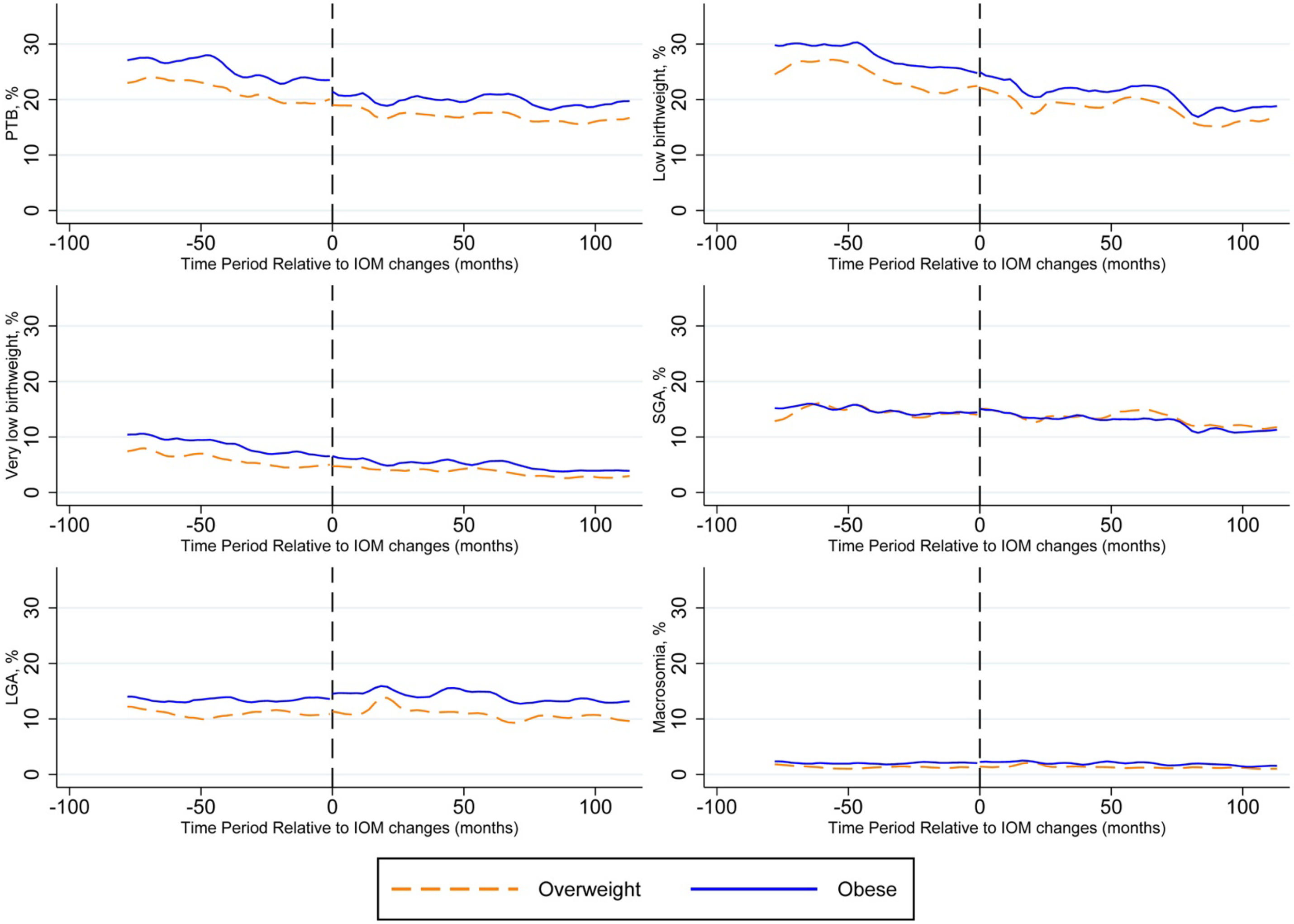

Note: Data drawn from the Pregnancy Risk Assessment Monitoring System (PRAMS). Time zero corresponds to July 1 2010.
Overweight (body mass index 25-29.9), obese (body mass index ≥30.0)
Abbreviations: IOM, Institute of Medicine; LBW, low birthweight; PTB, preterm birth; VLBW, very low birthweight

eFigure 3. Graphs of Trends for GWG Within, Above, or Below 1990 IOM Guidelines

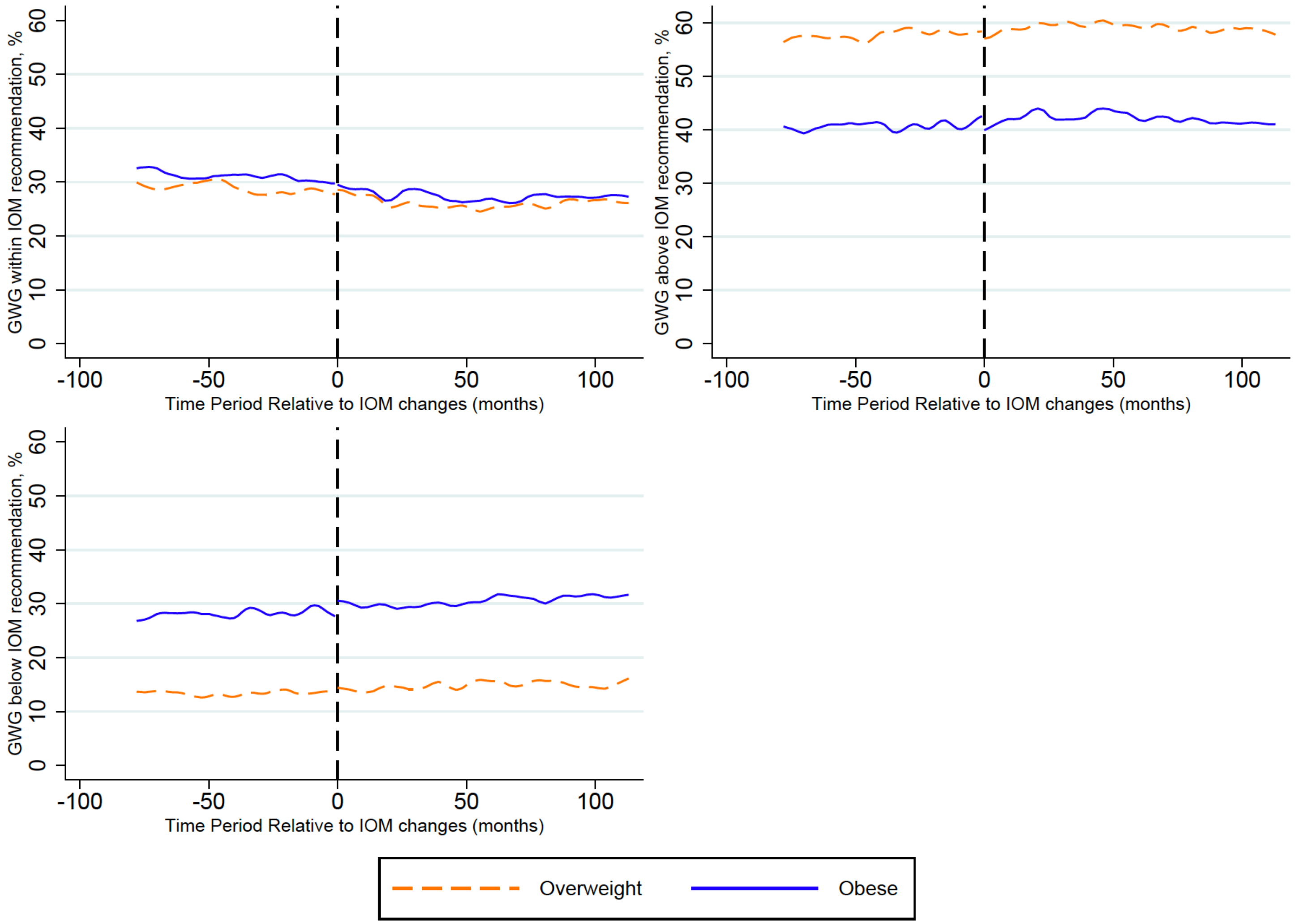

Note: Data drawn from the Pregnancy Risk Assessment Monitoring System (PRAMS). Time zero corresponds to July 1 2010. Overweight (body mass index 25-29.9), obese (body mass index ≥30.0)
Abbreviations: GWG, gestational weight gain; IOM, Institute of Medicine

eFigure 4. Effect of IOM Revised Guidelines on Outcomes by Education

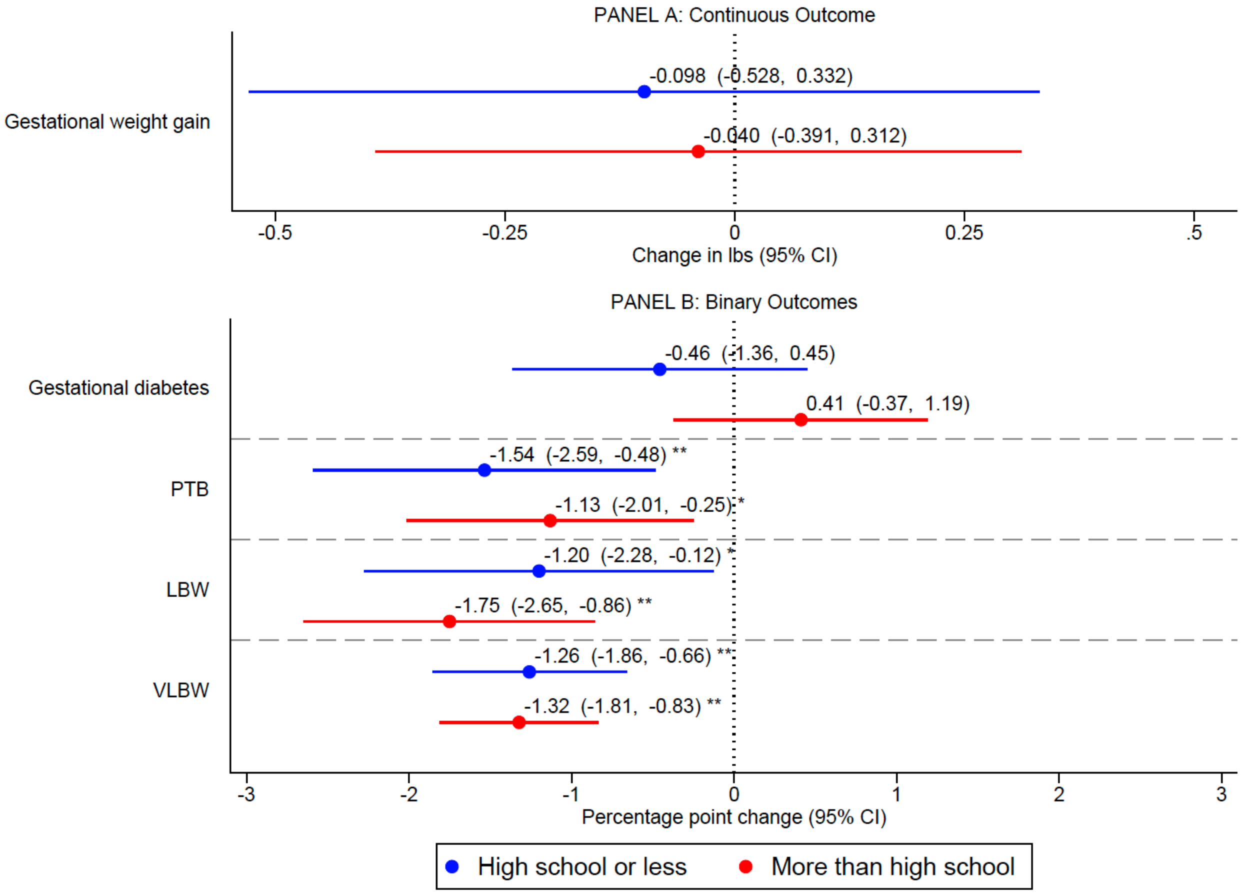

*p<0.05, ** p<0.01 indicates that a given estimate is statistically significantly different from zero.

Note: Data drawn from the Pregnancy Risk Assessment Monitoring System (PRAMS). Values represent the coefficients on the interaction term between a binary variable for whether a pregnancy occurred during the post-period (i.e., on or after July 2010) and a binary variable for whether a woman's pre-pregnancy BMI was categorized as overweight versus obese, stratified by education. Coefficients for binary outcomes were multiplied by 100 and therefore represent a change in percentage points. Analysis involved multivariable linear models (i.e., linear probability models for binary outcomes). Covariates included women's age, race/ethnicity, marital status, insurance for prenatal care, parity, household income in the year prior to delivery, and birth year. Sample was drawn from PRAMS participating states from 2004-2019 and included women with live-born singleton births with a gestational age of 20-44 weeks at delivery and whose pre-pregnancy weight was categorized as obese (BMI ≥ 30.0) or overweight (BMI 25.0-29.9).

Abbreviations: BMI, body mass index; CI, confidence interval; IOM, Institute of Medicine; PRAMS, Pregnancy Risk Assessment Monitoring System; PTB, preterm birth; LBW, low birthweight; VLBW, very low birthweight.

eFigure 5. Effect of IOM Revised Guidelines on Outcomes by Race/Ethnicity

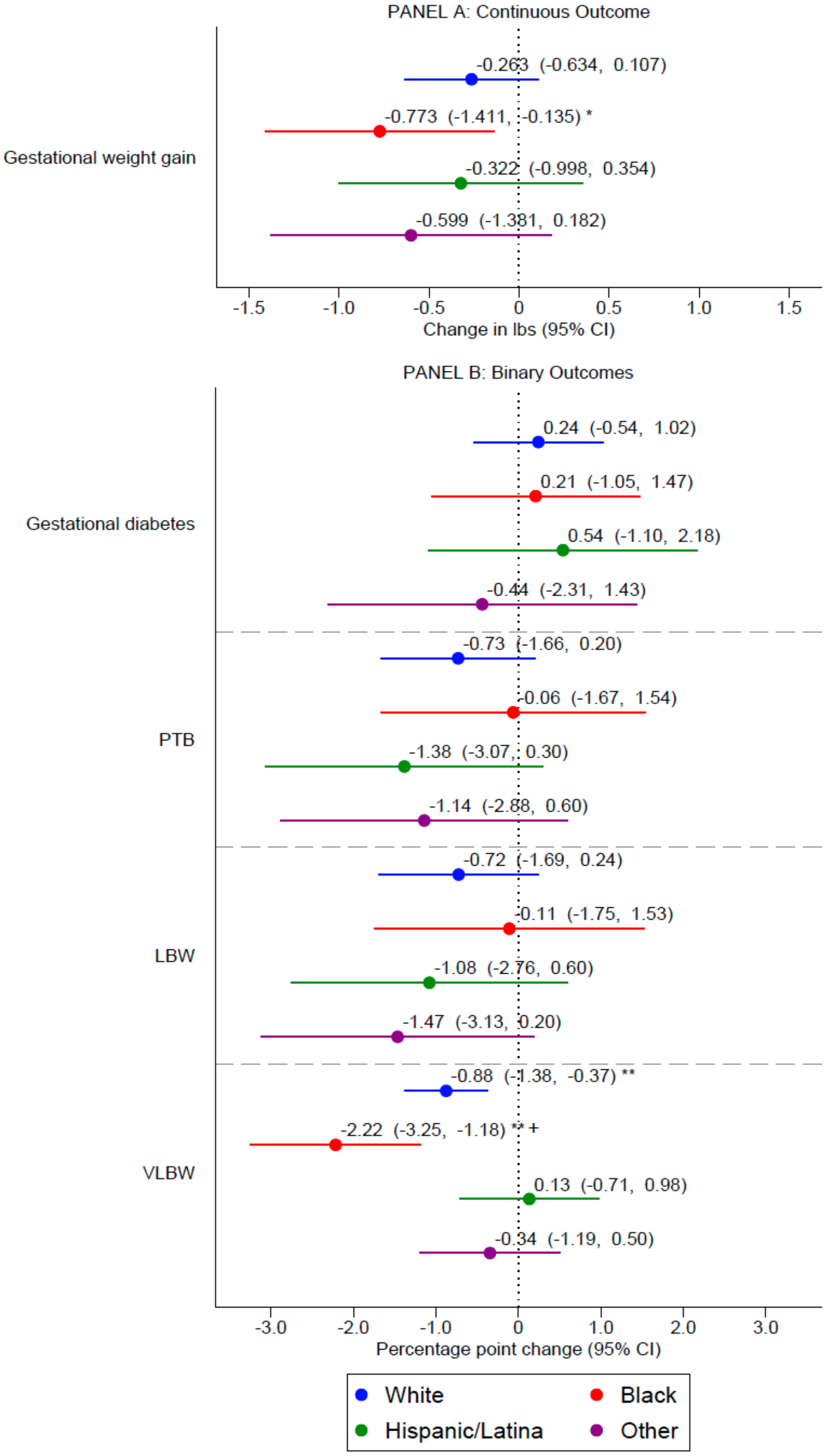

*p<0.05, ** p<0.01 indicates that a given estimate is statistically significantly different from zero.
+ p<0.05 indicates that a given estimate is statistically significantly different from the estimate for White women (the reference group) in regressions that included an interaction term for race/ethnicity.

Note: Data drawn from the Pregnancy Risk Assessment Monitoring System (PRAMS). Values represent the coefficients on the interaction term between a binary variable for whether a pregnancy occurred during the post-period (i.e., on or after July 2010) and a binary variable for whether a woman's pre-pregnancy BMI was categorized as overweight versus obese, stratified by race/ethnicity. Coefficients for binary outcomes were multiplied by 100 and therefore represent a change in percentage points. Analysis involved multivariable linear models (i.e., linear probability models for binary outcomes). Covariates included women's age, education, marital status, insurance for prenatal care, parity, and household income in the year prior to delivery, and birth year. Sample was drawn from PRAMS participating states from 2004-2019 and included women with live-born singleton births with a gestational age of 20-44 weeks at delivery and whose pre-pregnancy weight was categorized as obese (BMI ≥ 30.0) or overweight (BMI 25.0-29.9).
Abbreviations: BMI, body mass index; IOM, Institute of Medicine; PRAMS, Pregnancy Risk Assessment Monitoring System; PTB, preterm birth; LBW, low birthweight; VLBW, very low birthweight.

eFigure 6. Effect of IOM Revised Guidelines on Outcomes by Age

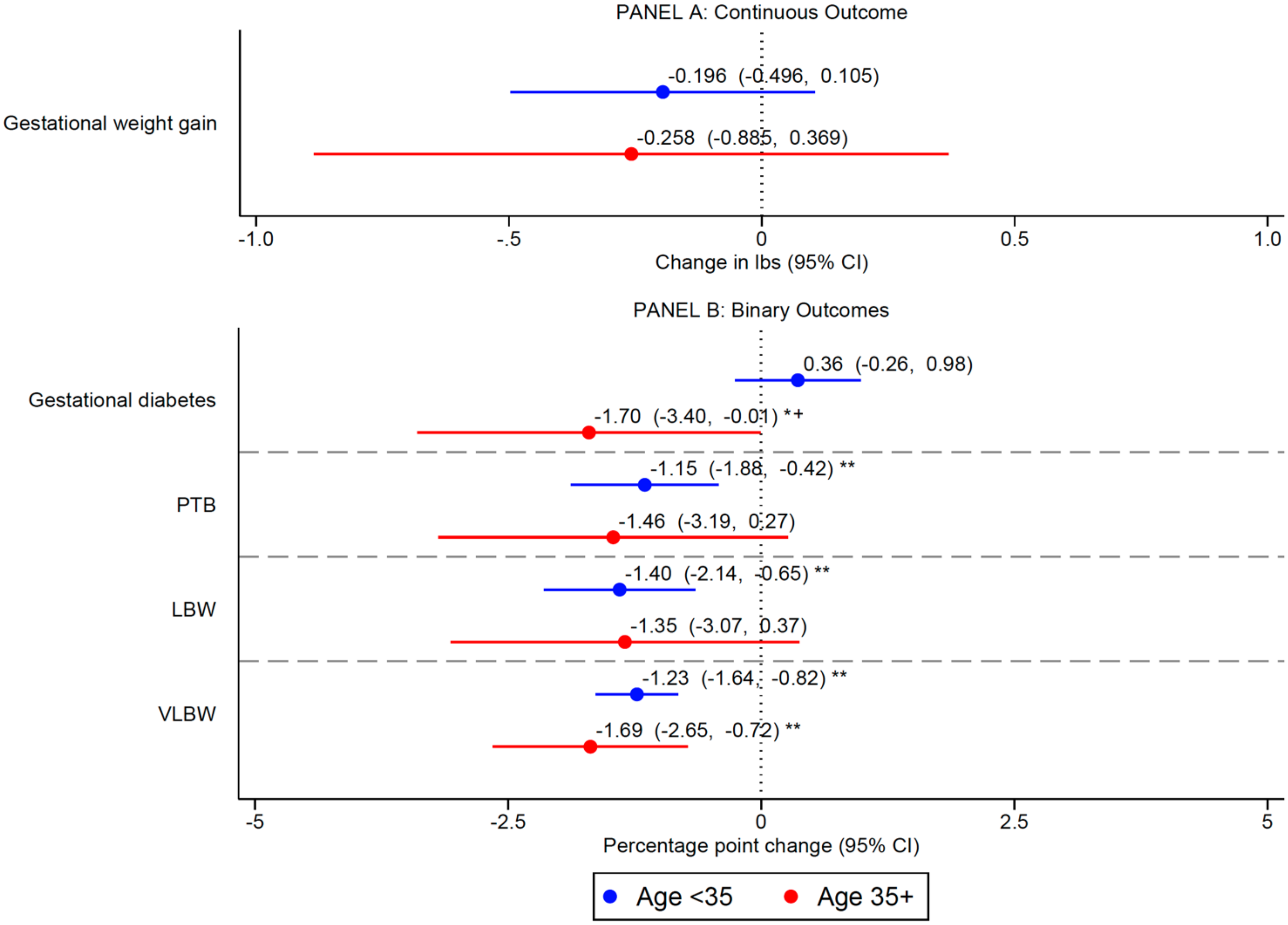

*p<0.05, ** p<0.01 indicates that a given estimate is statistically significantly different from zero.
+ p<0.05 indicates that a given estimate is statistically significantly different from the estimate for women under 35 years of age (the reference group) in regressions that included an interaction term for age.
Note: Data drawn from the Pregnancy Risk Assessment Monitoring System (PRAMS). Values represent the coefficients on the interaction term between a binary variable for whether a pregnancy occurred during the post-period (i.e., on or after July 2010) and a binary variable for whether a woman's pre-pregnancy BMI was categorized as overweight versus obese, stratified by age. Coefficients for binary outcomes were multiplied by 100 and therefore represent a change in percentage points. Analysis involved multivariable linear models (i.e., linear probability models for binary outcomes). Covariates included women's race/ethnicity, education, marital status, insurance for prenatal care, parity, and household income in the year prior to delivery, and delivery year. Sample was drawn from PRAMS participating states from 2004-2019 and included women with live-born singleton births with a gestational age of 20-44 weeks at delivery and whose pre-pregnancy weight was categorized as obese (BMI ≥ 30.0) or overweight (BMI 25.0-29.9).
Abbreviations: BMI, body mass index; IOM, Institute of Medicine; PRAMS, Pregnancy Risk Assessment Monitoring System; LBW, low birthweight; PTB, preterm birth; VLBW, very low birthweight.

eFigure 7. Effect of IOM Revised Guidelines on Outcomes by Parity

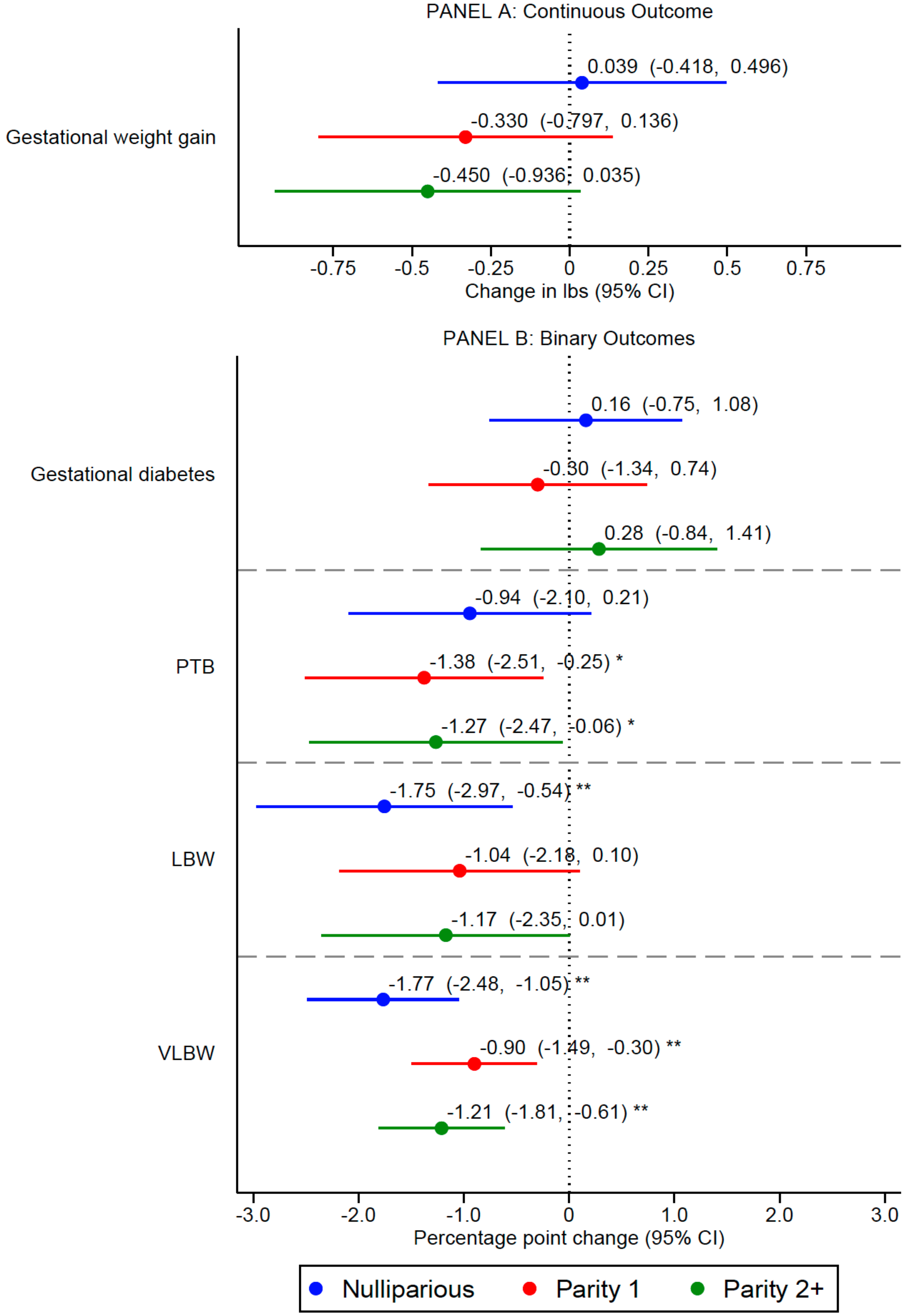

*p<0.05, ** p<0.01 indicates that a given estimate is statistically significantly different from zero.

Note: Data drawn from the Pregnancy Risk Assessment Monitoring System (PRAMS). Values represent the coefficients on the interaction term between a binary variable for whether a pregnancy occurred during the post-period (i.e., on or after July 2010) and a binary variable for whether a woman's pre-pregnancy BMI was categorized as overweight versus obese, stratified by education. Coefficients for binary outcomes were multiplied by 100 and therefore represent a change in percentage points. Analysis involved multivariable linear models (i.e., linear probability models for binary outcomes). Covariates included women's age, race/ethnicity, education, marital status, insurance for prenatal care, and household income in the year prior to delivery, and delivery year. Sample was drawn from PRAMS participating states from 2004-2019 and included women with live-born singleton births with a gestational age of 20-44 weeks at delivery and whose pre-pregnancy weight was categorized as obese (BMI ≥ 30.0) or overweight (BMI 25.0-29.9).
Abbreviations: BMI, body mass index; IOM, Institute of Medicine; PRAMS, Pregnancy Risk Assessment Monitoring System; LBW, low birthweight; PTB, preterm birth; VLBW, very low birthweight.

eFigure 8. Effect of IOM Revised Guidelines on Outcomes by WIC Status

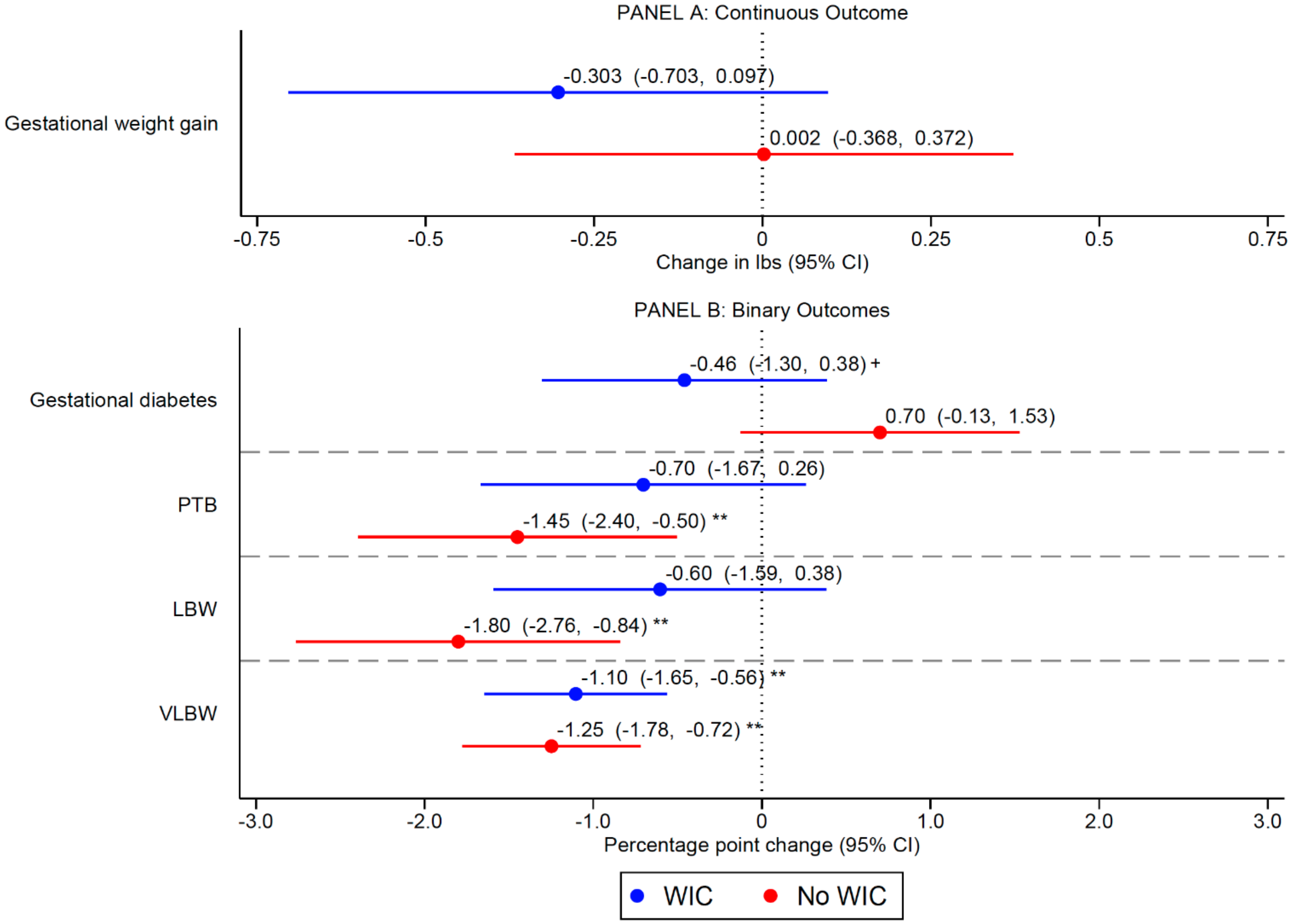

*p<0.05, ** p<0.01 indicates that a given estimate is statistically significantly different from zero.
+ p<0.05 indicates that a given estimate is statistically significantly different from the estimate for women with no WIC (the reference group) in regressions that included an interaction term for WIC receipt during pregnancy.
Note: Values represent the coefficients on the interaction term between a binary variable for whether a pregnancy occurred during the post-period (i.e., on or after July 2010) and a binary variable for whether a woman's pre-pregnancy BMI was categorized as overweight versus obese, stratified by WIC receipt. Coefficients for binary outcomes were multiplied by 100 and therefore represent a change in percentage points. Analysis involved multivariable linear models (i.e., linear probability models for binary outcomes). Covariates included women's age, race/ethnicity, education, marital status, insurance for prenatal care, and household income in the year prior to delivery, and delivery year. Sample was drawn from PRAMS participating states from 2004-2019 and included women with live-born singleton births with a gestational age of 20-44 weeks at delivery and whose pre-pregnancy weight was categorized as obese (BMI ≥ 30.0) or overweight (BMI 25.0-29.9).
Abbreviations: BMI, body mass index; IOM, Institute of Medicine; PRAMS, Pregnancy Risk Assessment Monitoring System; LBW, low birthweight; PTB, preterm birth; VLBW, very low birthweight; WIC, Special Supplemental Nutrition Program for Women, Infants, and Children.
